# Hospitals and Approved Societies

**Published:** 1923-12

**Authors:** S. Dunstan

**Affiliations:** House Governor and Secretary. Royal Victoria Infirmary, Newcastle-upon-Tyne


### CORRESPONDENCE.

[Correspondence on all subjects is invited, but we cannot be responsible for the opinions expressed by our correspondents, who must give name and address as a guarantee of good faith, but not necessarily for publication. Correspondents are reminded that conciseness greatly facilitates early insertion.]

### Hospitals and Approved Societies.

*To the Editor of* The Hospital and Health Review.

Sir,—At the present time there appears to be a keen interest and a general controversy concerning the National Health Insurance Act and the benefits to insured persons. The Minister of Health stated a few weeks ago that he wanted "the best medical service that could be got." May I venture to ask how this service is being supplied .at the present time? Is it not mainly supplied by the Voluntary Hospitals? I suggest that after ten years the position is sufficiently strong to justify the Voluntary Hospitals taking a definite stand as an integral or vital part in the health machinery of the community, without which it would be impossible for the Insurance Act to function.

After all, the Insurance Act only provides for such treatment as can be undertaken by a General Practitioner, there being no provision made for clinical investigation or other facilities to assist in the diagnosis of disease and consequent treatment. The greatest work, then, for the health of the community has to be carried out in our Voluntary Hospitals which are provided with all the latest and most up-to-date equipment, and served by the most experienced and skilled men that the medical profession can produce, without the slightest remuneration or consideration by way of return.

The Minister of Health in 1920 endeavoured to induce the Approved Societies to make grants from their surplus funds to the Voluntary Hospitals towards the maintenance cost of their members, and gave legislative sanction, but out of the thousands of Approved Societies only a few agreed to comply with the Minister's request. In addition to this, the Approved Societies were strongly recommended by the Cave Commission to help the Voluntary Hospitals. We are now in a position to state the volume of work done here and the expenditure involved during 1922 for Panel Patients treated, all of whom were members of Approved Societies under the National Health Insurance Act.

*Statement showing Patients treated in the Royal Victoria Infirmary during the year* 1922.

IN-PATIENTS.

| Members of— | | Members. | Days in Hospital. | At 8/4 per day. |
|---|---|---|---|---|
| 1. Contributing Societies for a certain number of whom a grant has been received from Surplus Fund | .. .. .. | 684 | 12,080 | £5,033 6 8 |
| 2. Contributing Societies (no grant) | .. | 376 | 5,670 | £2,362 10 0 |
| | | 1,060 | 17,750 | £7,395 16 8 |
| 3. Non-Contributing Societies | .. | 2,429 | 43,501 | £18,125 8 4 |
| Total | .. | 3,489 | 61,251 | £25,521 5 0 |
| OUT-PATIENTS. | .. | 28,520 | at 2s. 6d. | 3,565 0 0 |
| Total | .. | 32,009 | | £29,086 5 0 |

As regards No. 1, the sum of £2,230 11s. has been received. No. 2 relates to patients who, after repeated applications, either declined to give the necessary information as to their Society and membership number, or gave erroneous replies. In consequence no claim could be submitted. No. 3 represents the great bulk of Approved Societies who decline to make any grant from their surplus funds to Voluntary Hospitals.

Roughly, one would be justified in stating that one-third of cases treated are patients insured under the National Health Insurance Scheme. The Panel System has undoubtedly been the means of enabling patients to seek earlier medical advice, whereas, before the Act, advice was often only sought when a patient was beyond recovery.

Considering that medical science has been the means of increasing the expectation of life by about ten years since 1903, and that the death rate has been reduced from 1.8 to 1.2 per cent., it would appear that a more generous recognition should be given by the life insurance companies to the Voluntary Hospitals, especially those attached to Medical Schools, where very expensive accommodation and equipment must be provided for investigation, research, and other methods which tend to help in the diagnosis and better treatment or prevention of disease.

S. Dunstan,
House Governor and Secretary.
Royal Victoria Infirmary, Newcastle-upon-Tyne.

